# Applications of mass spectrometry imaging in botanical research

**DOI:** 10.1007/s44307-024-00014-y

**Published:** 2024-02-15

**Authors:** Yi-Jun Chen, Hai-Sheng Zeng, Hong‑Lei Jin, Hong‑Bin Wang

**Affiliations:** 1 State Key Laboratory of Traditional Chinese Medicine/School of Pharmaceutical Sciences, Guangzhou University of Chinese Medicine, Guangzhou, 510006 China; 2 Guangdong Provincial Kye Laboratory of Prescription and Syndrome, Guangzhou, 510006 China; 3 Pharmaceutical Department, The Affiliated Hospital of Youjiang Medical University for Nationalities, Baise, 533000 China; 4 Guangzhou Key Laboratory of Chinese Medicine Research On Prevention and Treatment of Osteoporosis, The Third Affiliated Hospital of Guangzhou University of Chinese Medicine, Guangzhou, 510006 China; 5 Key Laboratory of Chinese Medicinal Resource From Lingnan, (Guangzhou University of Chinese Medicine), Ministry of Education, Guangzhou, 510006 China

**Keywords:** MSI, MALDI-MSI, DESI-MSI SIMS Botany

## Abstract

Mass spectrometry imaging (MSI) serves as a valuable tool enabling researchers to scrutinize various compounds, peptides, and proteins within a sample, providing detailed insights at both elemental and molecular levels. This innovative technology transforms information obtained from a mass spectrometer— encompassing ionic strength, mass-to-charge ratio, and ionized molecule coordinates—within a defined region into a pixel-based model. Consequently, it reconstructs the spatial distribution of ions, allowing for a comprehensive understanding of molecular landscapes. The significance of MSI lies in its ability to offer multiple advantages, including straightforward sample preparation and remarkable sensitivity, all achieved without the necessity for labeling. Particularly in the realm of plant biology, MSI finds frequent application in examining the distribution of target metabolites and other components within plant tissues. This review delves into the fundamental principles, distinguishing features, merits, and applications of three prominent MSI technologies. Furthermore, we aim to assist readers in navigating the utilization of MSI in their plant biology research by discussing primary challenges, proposing potential solutions, and elucidating future prospects associated with this cutting-edge technology.

## Introduction

Plants play important roles in the daily lives of people, serving as major sources of food and medicines. Therefore, scientists have long-since studied botany. Plants contain an intricate metabolic network that produces diverse metabolites such as polyphenols, alkaloids, and terpenoids in a spatially and temporally defined manner. These metabolites have crucial functions in plant disease resistance, defense responses, and signal transduction, and many have important uses as pharmaceutical compounds. Conventional metabolomics technologies are useful for elucidating the connections between the biosynthesis and transport of bioactive compounds in plants, as well as the relationship between the accumulation of secondary metabolites and environmental conditions. However, current research techniques are not sufficient to fully elucidate the unknown mechanisms underlying the transport and metabolism of components being tested in botanical research, hampering the precise improvement of plant quality.

In 1997, Caprioli et al. introduced mass spectrometry imaging (MSI) as an innovative molecular imaging technique to investigate the spatial distribution of peptides and proteins in biological tissue samples (Caprioli et al. 1997). This technique uses various ionization probes with distinct principles and structures to scan a sample, enabling in situ desorption/ionization of the target substance within the sample. The desorbed/ionized substance is analyzed in a mass spectrometer, generating a set of mass spectrograms that correspond with the spatial position of the sample. Mass imaging software then processes the set of mass spectra to generate tissue distribution images for each mass-to-charge (*m*/*z*) ratio. With the development of MSI technology, the spatial distribution of analytes in plants could be efficiently presented, allowing visual analysis of plant internal components. MSI analysis provides an effective tool for studying processes such as the biosynthesis, metabolism, and transport of substances in plants, plant self-regulation, and interactions between plants and the environment in a more in-depth and systematic manner. Moreover, MSI does not require immunofluorescence labeling or complex sample pretreatment. These advantages make MSI suitable for a wide range of applications, including analyzing rhizomes and other structures, leaf surfaces, plant hormones, the pathophysiology of fruits and vegetables, the molecular networks of natural products, as well as plant and microbial symbiosis and interactions.

Ongoing advancements in MSI technology, along with innovations to meet the diverse needs of various research disciplines, have led to the development of numerous MSI technologies. Three MSI techniques have gained prominence for use in plants: matrix-assisted laser desorption ionization–mass spectrometry imaging (MALDI-MSI) (Mi et al. 2020; Stoeckli et al. 2001), desorption electrospray ionization–mass spectrometry imaging (DESI-MSI) (Cooks et al. 2006), and secondary ion mass spectrometry (SIMS) imaging (Q. B. Liu et al. 2022; Todd et al. 2001). The application of these MSI techniques to phytochemistry research has revealed the spatial distributions of various secondary metabolites in plant tissues and laid the foundation for further in-depth studies using MSI techniques in plant research.

Here, were present the basic principles, technical characteristics, advantages, and disadvantages of different MSI technologies. We also discuss the application of each technology in plant breeding and for analyzing plant growth and development, plant quality, and stress resistance. Our aim is to describe the development of MSI technology for use in botanical research and to help readers choose the most appropriate MSI method based on their research objectives.

## Overview of the basic principles and operational procedures of MSI

In recent decades, there has been rapid progress in the development of MSI technology, with a particular emphasis on improving the ionization mode and mass analyzer. Based on advances in ionization methods, SIMS was initially employed for solid plane analysis in the 1960s (Gilmore et al. 2019). Subsequently, MALDI and electrospray ionization (ESI) were introduced as alternatives. Several advances have been made in atmospheric pressure in-situ MSI technology, such as direct analysis in real time (DART), nanostructure-initiator mass spectrometry (NIMS), and the atmospheric pressure solid analysis probe (ASAP) (Moser et al. 2022; Yun et al. 2021). Numerous mass spectrometry techniques are currently available to complement MSI technology, including time-of-flight mass spectrometry (TOF–MS), ion trap mass spectrometry (IT-MS), quadrupole time-of-flight mass spectrometry (Q-TOF–MS), and Fourier transform ion cyclotron resonance mass spectrometry (FTICR-MS). Using MSI technology for non-targeted in situ analysis of surface molecules in biological samples enables researchers to establish an association between the metabolic characteristics of the samples under analysis and their histological and morphological characteristics, making MSI technology useful for plant biology (Baker et al. 2017).

**Fig. 1:**
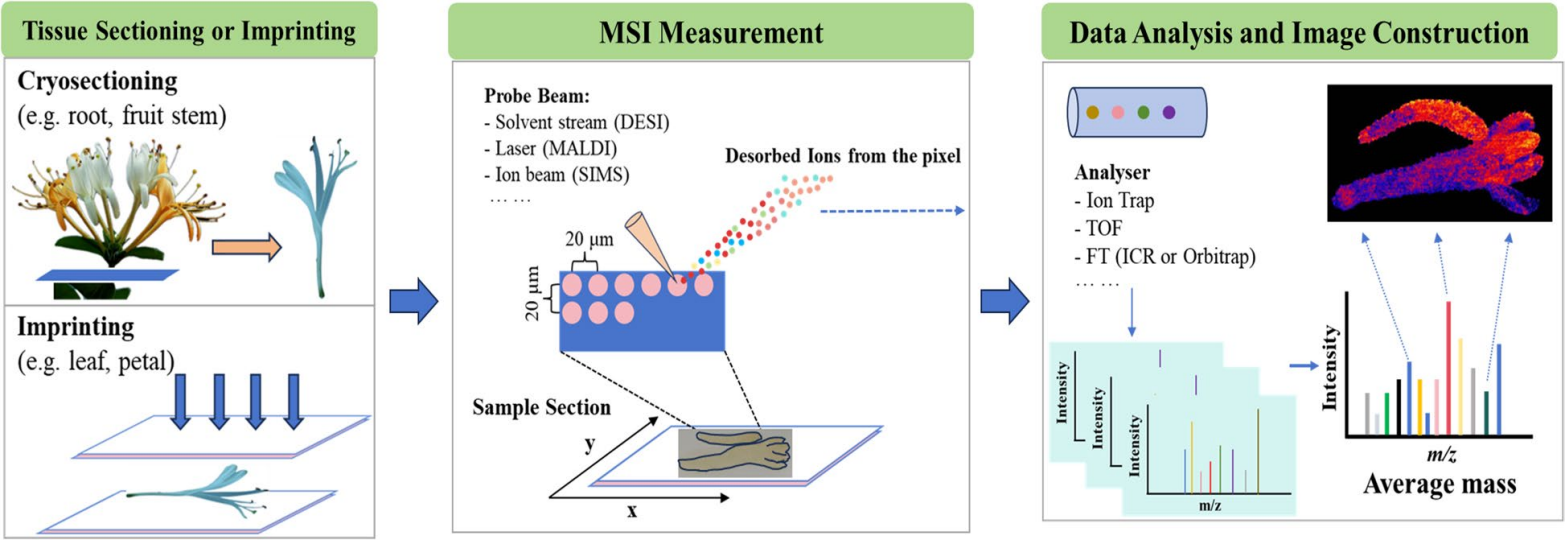
Typical workflow of MSI of plant tissues

Mass spectrometry can obtain qualitative and quantitative information by collecting the *m*/*z* ratios of compounds, allowing two fundamental questions to be explored: What is the nature of the substance, and how many compounds are contained in the substance itself? Imaging technology utilizes some in situ ionization sources associated with mass spectrometry and visual imaging software, allowing *m*/*z* ratios to be detected based on different signal colors and intensities, providing information about the locations of the compounds. Hence, from a technical standpoint, MSI technology is an analytical approach used to observe plant samples by assessing the *m*/*z* values of gaseous ions (Fig. 1). Initially, the sample undergoes ionization. A mass spectrum is then acquired by distinguishing ions based on their respective motion behaviors within electric or magnetic fields. The mass spectrum and associated data facilitate qualitative and quantitative analyses of the sample. Subsequently, the acquired data can be analyzed using MSI software, enabling the precise localization of various molecules within the sample. Furthermore, the molecular structure and spatial distribution of the target compound can be accurately characterized without the need for any labeling agents. This approach has notable advantages, such as high sensitivity, accuracy, reliability, and rapid analysis.

To better understand the mysteries of living organisms, scientists have developed a variety of imaging methods to view their internal structures, such as optical microscopy (OM) (Balasubramanian et al. 2023), electron microscopy (EM) (Lewczuk & Szyryńska 2021), fluorescence imaging (FI) (Lauwerends et al., 2021), magnetic resonance imaging (MRI) (Thomas et al. 2023), atomic force microscopy (AFM) (Q. Liu et al. 2023), and scanning tunnel microscopy (STM) (Duan & Xu 2023). Although these techniques have been very useful in medicine and science, they have many several shortcomings. The resolution of commonly used medical imaging methods is relatively low, such as “MRI is less reliable at detecting tumours that are small (< 0.5 cc), low grade (Gleason score 6) or in the transitional zone” (Thompson et al. 2013), which has greatly limited their application for medical detection. By contrast, although FI technology has higher resolution, it requires use of fluorescent labeling, which alters the physical and chemical environment of the target analyte and could potentially generate errors for clinical applications (Chu et al. 2023). Although the rapidly developing AFM and STM techniques have solved the problem of low resolution, they still cannot be used to analyze the measured object qualitatively (Liu et al. 2020). In contrast to conventional imaging techniques like nuclear magnetic resonance and fluorescence labeling, MSI does not require specific labeling of samples and has the advantages of simple sample preprocessing steps and high spatial resolution. Moreover, MSI utilizes the efficiency of mass spectrometry to image the target object and provide structural information (H. Jiang et al. 2022; Nilsson et al. 2015; Spengler 2015).

Although the principles of different MSI methods are not exactly the same, they are based on determining *m*/*z* ratios combined with specific imaging software. The target molecules in different parts of the measured object are detected, identified, imaged, and superimposed, providing an image that reflects the spatial distribution of the content of the measured object. Compared with other imaging technologies, MSI has the following advantages: 1) relatively straightforward sample pretreatment that eliminates the need for fluorescence or isotope labeling; 2) rapid detection of tissue-specific markers at the molecular level; 3) simultaneous acquisition of information regarding the composition, spatial distribution, and abundance of various molecules within the tested tissue; 4) visualization throughout the entire process that facilitates low-loss or nondestructive detection, enabling qualitative and quantitative analysis at the same time; and 5) high detection sensitivity that enables the detection of trace amounts of a compound and the differentiation of molecular structure information among similar compounds (Y. Jiang et al. 2022; Nilsson et al. 2015; Spengler 2015).

However, MSI also has some shortcomings, such as the following: 1) instrument complexity that requires professional operation and maintenance, as well as for the processing and interpretation of data; 2) limited spatial resolution that cannot reach the resolution at the cellular or subcellular level; 3) dependence on careful sample preparation to prevent signal distortion or false positive or negative results. Therefore, an in-depth understanding of the characteristics and advantages of existing MSI technology is essential for its effective and efficient application in botanical research. In the following chapters, we discuss three MSI techniques that are widely used in botanical research.

### MALDI-MSI

In 1988, Karas & Hillenkamp successfully employed laser ionization to identify macromolecular compounds with molecular weights reaching from 10 to 34 kDa, thereby giving rise to MALDI technology and enabling the detection of biological macromolecules through mass spectrometry (Karas & Hillenkamp 1988). In the 1990s, Spengler et al. and Caprioli et al. designed methods for the visualization of multiple components on the tissue surface, which was successfully used in the field of molecular imaging. Since then, this technique has received great attention and has developed rapidly, becoming one of the most commonly used MSI techniques (Caprioli et al. 1997). MALDI-MSI can be categorized into vacuum MALDI-MSI and atmospheric MALDI (AP-MALDI). Vacuum MALDI involves placing the dried sample into a high-vacuum cavity, whereas in AP-MALDI, the sample is placed directly in a normal-pressure environment. Subsequently, the substance to be analyzed is combined with a matrix capable of absorbing ultraviolet or infrared wavelengths, resulting in co-crystallization. Laser irradiation then causes the desorption and ionization of the matrix molecules, enabling in situ ionization of the substance to be measured. AP-MALDI is simpler to perform than vacuum MALDI, and it helps increase ion stability and reduce compound cracking, but its sensitivity is relatively low, making vacuum MALDI more common.

**Fig. 2:**
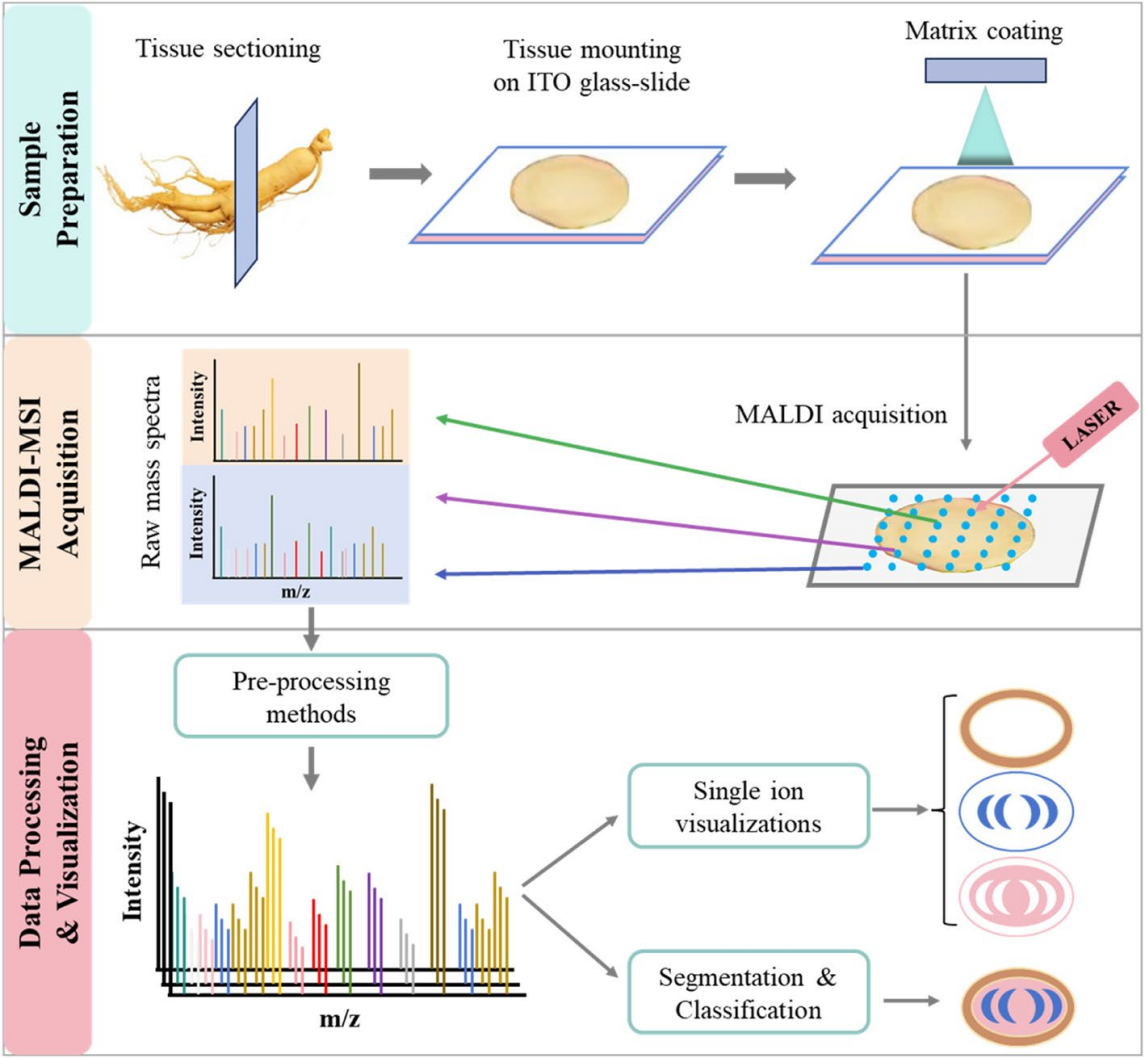
Workflow for a typical matrix-assisted laser desorption/ionization–mass spectrometry imaging (MALDI-MSI) experiment

Initially, MALDI was primarily employed to examine macromolecules, specifically proteins (Karas & Hillenkamp 1988). As MALDI technology has progressed, however, particularly with the emergence of MALDI-MSI technology, its application has expanded to encompass the analysis of lipids, small metabolites, and small bioactive molecules in their native environments (Gupta et al. 2019; Kompauer et al. 2017; Sarabia et al. 2018; X. Wang et al. 2022). MALDI-MSI is currently the most widely used MSI technique. Research on the distribution patterns and accumulation mechanisms of plant secondary metabolites has attracted widespread attention (K. He et al. 2021). Plants show major variations in the distribution patterns and accumulation mechanisms of secondary metabolites among different stages of growth and in different tissues and organs (Montini et al. 2020). Consequently, researchers have been exploiting the advantages of MALDI-MSI to investigate the biosynthesis, transport, and accumulation of plant secondary metabolites. Due to the unique composition and structure of plant tissues, the sample preparation and analysis methods are more stringent than for animal tissues. The procedure for MALDI-MSI of plant tissues (shown in Fig. 2) consists of three major steps: sample preparation, mass spectrometry data acquisition, and data processing and visualization.

Sample preparation is important for achieving high-quality mass spectrometry images. Factors such as the tissue sampling method, sample integrity, sample thickness, matrix selection, and spraying technique substantially affect the outcome of MALDI-MSI (Rahman 2009). In contrast to animal tissues, which typically contain abundant levels of fat, plant samples are characterized by their firm texture, high water content, and abundance of fiber in the cell wall. Consequently, preparing frozen sections of plants tissues is more demanding compared to animal tissue. Frozen sectioning is widely employed for sample preparation for MALDI-MSI (Nguyen et al. 2017). Plant samples possess a cell wall structure with substantial intercellular space and high water content. Hence, embedding is usually essential for preserving the structural integrity of tissues, thereby limiting the occurrence of wrinkles and fragmentation in sliced samples, ultimately enhancing the quality of frozen sections. The widely employed embedding agent OCT (optimal cutting temperature) compound is unsuitable for MALDI-MSI due to issues with sample contamination and ion inhibition. Carboxymethyl cellulose, gelatin, and ice are extensively used as embedding agents, with agent selection contingent upon the unique characteristics of the sample under investigation (Y. He et al. 2019; Keller et al. 2020). The sensitivity of MALDI-MSI is strongly affected by the thickness of the tissue slices; thus, it is imperative to regulate tissue thickness while preserving structural integrity. To guarantee laser precision and the strength of signal output during analysis, slices are typically maintained at a thickness of around 20–50 μm for examining secondary metabolites in plant tissues. Analyzing thin samples that cannot be embedded, such as leaves and petals, requires the use of molecular imprinting, in which samples are transferred onto a flat surface using techniques such as flat pressing and subjected to MSI analysis (Dutkiewicz et al. 2021).

Additionally, when preparing frozen sections, it is crucial to prevent enzymatic degradation and compound displacement. Therefore, it is customary to rapidly freeze fresh samples using liquid nitrogen or dry ice, followed by cutting frozen sections. If the prepared sample needs to be stored briefly, it can be freeze-dried and wrapped in aluminum foil before being placed in a sealed container with desiccant. However, for long-term storage, it is advisable to store the sample at –80℃. While this storage approach can minimize changes in the composition and quantity of components, it remains challenging to entirely prevent cellular shrinkage due to water loss from plant tissues with high water content (Seeley et al. 2008).

During sample analysis by MALDI-MSI, it is necessary to mix the sample solution with excess matrix solution and drop it onto the target plate. After the solvent evaporates, the sample and matrix form co-crystals. Then, under the irradiation of a fixed-wavelength pulsed laser, the laser energy is transferred to the matrix molecules. After the matrix molecules gain laser energy, they undergo energy level transition and enter the excited state. Next, the matrix molecules act as proton donors or proton acceptors, and charge transfer occurs between the sample molecules. This leads to the desorption and ionization of the sample molecules, which are then vaporized and released into the mass analyzer. During this process, some of the matrix molecules are also ionized along with the sample molecules and are subsequently analyzed (Cornett et al. 2007; Maddalo et al. 2005). Therefore, the type of matrix has an important influence on sample detection and should be selected based on the purpose of the experiment. In general, the ideal matrix should have the following characteristics: 1) does not interact with the analyte; 2) strong laser energy absorption; 3) stable properties (does not easily change during the detection process); 4) good compatibility with the analyte and ability to form a uniform co-crystal; and 5) low steam pressure and easily undergoes sublimation (Huang et al. 2022).

Traditional MALDI-MSI matrixes are organic molecules that are often used in combination with the properties of analytes. When small organic acids are used as the matrix, they exhibit strong proton transfer with the molecules to be measured, thereby producing strong mass spectrometry signals. However, these small organic acids produce a lot of matrix background interference in the low-molecular-weight region, thus inhibiting or covering the ion peak of the object to be measured. Therefore, a small-molecule organic acid matrix is more suitable for the analysis of biological macromolecules such as sugars, proteins, peptides, and lipids (Seeley et al. 2008). In recent years, scientists have developed many new organic matrixes that can be used to detect small-molecule compounds, such as N-butyl-4-hydroxy-1,8-naphthalimide (BHN) and N-phenyl-2-naphthylamine (PNA). Such matrixes have greater laser energy absorption capacity, lower background interference, and better vacuum stability. However, the existing organic matrixes still do not completely avoid the occurrence of “coffee rings”, i.e., the formation of non-uniform co-crystals on the surface of the target plate between the matrix and the analyte. This situation can lead to poor reproducibility and reduced sensitivity of the mass spectrum signal. Therefore, scientists are continuing to develop new organic matrixes to help overcome these shortcomings (Xu et al. 2018).

Unlike organic matrixes, inorganic matrixes often produce low background signals and exhibit high performance. These inorganic matrixes primarily include inorganic nanoparticles or substances with nanostructures. Five major types of inorganic matrixes are commonly used: carbon-based matrixes, silicon matrixes, metal nanoparticle and oxide matrixes, composite material matrixes, and non-metallic semiconductor matrixes. However, whether inorganic nanomaterial matrixes can effectively make up for the deficiencies of traditional organic matrixes in MALDI-MSI has not been fully explored, making the selection of application scenarios and the further development and improvement of these materials uncertain. In the future, the development of inorganic matrixes should focus improving the light absorption characteristics, strengthening the photothermal conversion efficiency, and enhancing their electronic conductivity (He et al. 2019; Shi & Deng 2016).

MALDI-MSI matrix spraying and crystallization are important steps in MSI that directly affect the sensitivity and reproducibility of this technique. Commonly used matrix coating techniques include the dry drop, spray, and sublimation methods. The dry drop method often leads to matrix diffusion, causing the matrix to be unevenly distributed on the substance to be measured, which reduces the repeatability of this method. Therefore, this method is rarely used for sample composition analysis via MALDI-MSI. The spray method includes the manual spray gun method and the ultrasonic spray method. The use of a manual spray gun is relatively simple but is readily affected by human factors, leading to non-uniform matrix spraying. Due to this issue, the automatic spray system is becoming increasingly popular. Ultrasonic spraying can make the distribution of the matrix more uniform and help enhance the signal strength of the metabolites. However, many commercially available automatic matrix sprayers are costly and their operation relatively complex (Cailletaud et al. 2018; Zubair et al. 2016).

Unlike the first two techniques, the sublimation method does not require organic solvents to be added during the process. Moreover, the matrix crystal particles formed by recrystallization are small with better uniformity, helping solve the problem of displacement of the object to be measured using the first two matrix spraying methods. However, due to the absence of a solvent, the interaction between the matrix and the substance under analysis is weakened. This may cause the spectral responses of some substances under analysis to decrease and can also lead to defects in spatial resolution and data reproducibility. These shortcomings could be solved by sublimation “recrystallization”, It should also be noted that the sublimation method requires the boiling point of the matrix to be lower than its melting point, making it unsuitable for some matrixes, thus limiting its scope of application (Cailletaud et al. 2018). Therefore, researchers should choose the appropriate matrix coating method based on the characteristics and specific requirements of their own sample detection.

Data analysis, the most important component of MSI, has a great impact on the final test results. In MSI analysis, each biological tissue slice may produce hundreds of gigabytes of raw imaging data, making it difficult to perform subsequent data analysis. To meet the needs of MSI data processing, it is important to develop specific software packages and select the appropriate data processing methods. The most common open-source software packages and free MSI platforms include MSIReader, OmniSpec, BioMap, and Datacube (Bennett & Fernandez 2015; Spraker et al. 2020; Sturtevant et al. 2021). Several personalized and commercial sample analysis computing platforms are used to analyze specific types of data, such as MassImage, pyBASIS, and M2aia. These platforms generate more refined clustering results for subsequent analysis by enabling interactive visualization, 3D image reconstruction, comparison and analysis of in situ information from tissue samples via machine learning, and extraction of advanced features from ion images (Cordes et al., 2021). Commonly used data processing methods can improve the quality of MSI images by removing noise, correcting *m*/*z* peak deviation, and performing normalization. The purpose of normalization is to reduce the signal differences between pixels, which may be caused by uneven substrate coating or surface ion suppression. Normalization based on total ion count (TIC), currently the most commonly used method, can improve the quality of MSI (Minami et al. 2007). However, for some compounds that are distributed in a very limited area and have high abundance, TIC homogenization can cause data distortion. In these cases, other homogenization methods should be considered (Pirman & Yost 2011). The above drawbacks could be mitigated by using suitable internal standards in MSI targeted analysis to complete the normalization processing of the MSI data to be tested, such as “an internal standard correction strategy” (W. Tang et al. 2019), thereby improving the stability of data quality. This technique can also be used for semi-quantitative and quantitative MSI analysis.

In addition to data normalization, principal component analysis (PCA), partial least squares-discriminate analysis (PLS-DA), orthogonal partial least squares discriminant analysis (OPLS-DA), and cluster analysis are also widely used for processing imaging data. When using PCA, when the difference between groups is small but the difference within groups is large, it is difficult to draw a correct conclusion. PLS-DA, which is based on partial least squares regression, can strengthen the differences between groups by artificially adding grouping variables. However, PLS-DA is not effective in processing data that are correlated with each other and unrelated to classification. By removing the independent variable X that is not associated with the categorical variable Y, the OPLS-DA analysis method mainly concentrates classification information into a single principle component. This makes the data model simpler and easier to interpret and its discrimination effect and visualization of the principal component score chart more obvious. Cluster analysis can perform homologous clustering of the data, and the resulting cluster graph can show the relationships between the samples in MSI research (Zamora Obando et al. 2021). MSI data analysis usually involves combining a variety of statistical analysis methods for data processing, which can more effectively extract and display the associations and differences between MSI data information.

### DESI-MSI

In 2004, Cooks developed the DESI ion source (Cooks et al. 2006), which was later used to examine the distribution properties of lipids in rat brains, gradually leading to its application to the in situ analysis of lipids and other constituents in animal tissues (Girod et al. 2011). DESI-MSI involves electrospray ionization to rasterize solvent droplets under atmospheric pressure and their direct desorption on the sample surface for rapid and less destructive chemical screening. The molecules in the tissue can be accurately observed at the highest resolution (at the μm scale), and the dataset provides a picture of each detected analyte. DESI-MSI analysis does not require complex sample preparation and has a simple workflow and rich functionality. The basic principle of DESI-MSI is the droplet extraction and sample desorption mechanism. In DESI-MSI, droplets of a high-speed ionized solvent desorb the analyte of interest directly from the sample surface, dissolving the compound into a thin film on the sample surface. To achieve chemical desorption, the solvent is electrosprayed through a transmitter capillary at high pressure, creating a charged “primary” droplet, which is atomized and directed to the sample. In this way, metabolites located on the surface of the sample are desorbed into gaseous “secondary” droplets, which transport molecular ions to the MS inlet, where *m*/*z* values are measured. Subsequently, depending on the polarity of the target metabolite, imaging can be performed using different spray solvents from the DESI source for the analysis of polar and nonpolar compounds (Ifa et al. 2010; Mamun et al. 2020). In DESI-MSI analysis, standard solvents (such as methanol and acetonitrile) are usually used, and 2% to 5% water is usually added. However, different types of solvents are used depending on specific needs and applications.

By contrast, with the typical MALDI-MSI, the sample is coated or co-crystallized with an absorbent substrate and irradiated with pulses from a UV or IR laser. The matrix absorbs the radiation, transfers the energy into the sample, and aids in its ionization. The laser desorption/ionization process typically also destroys the sample during MALDI-MSI. Since MALDI is usually performed under a vacuum, samples often must be freeze-dried prior to analysis, making these techniques incompatible with living tissue (Huang et al. 2022). DESI is considered to be the least destructive method for the ionization of living tissue samples, allowing it use in areas that other MSI methods cannot approach (Parrot et al. 2018).

**Fig. 3:**
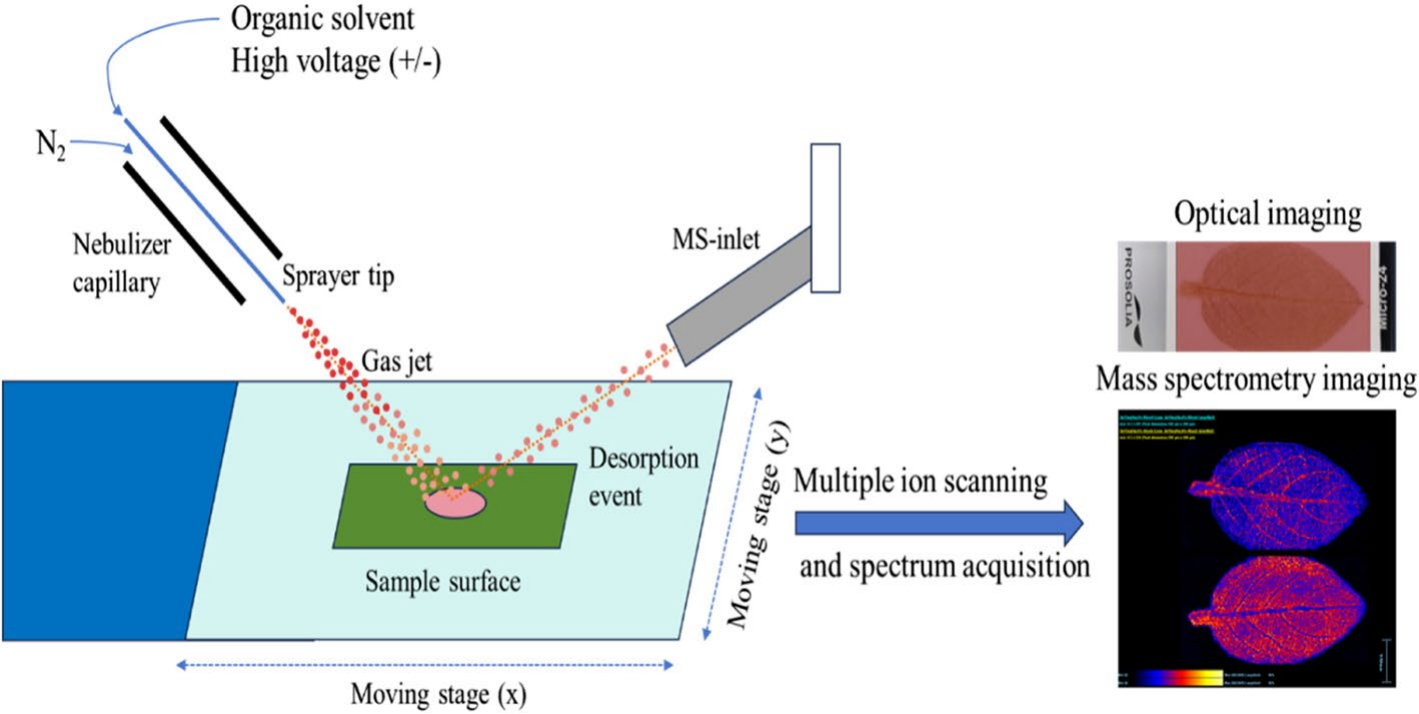
Schematic diagram of desorption electrospray ionization–mass spectrometry imaging (DESI-MSI)

Figure 3 illustrates the two-part structure of the capillary in the ion source used in DESI-MSI. The inner capillary is responsible for transporting the spray solvent, while the outer capillary conveys the atomizer. As described above, during sample analysis, the spray solvent is initially applied with a specific voltage, emitted from the inner sleeve of the atomizer, and swiftly atomized and accelerated by the high-speed nitrogen expelled from the outer sleeve of the atomizer. Consequently, charged droplets collide with the sample surface, causing the sample to transition into the gas phase after being hit by the high-speed droplets. Due to nitrogen purging and drying, the charged droplets carrying the sample undergo desolvation and travel through the ion transport tube at atmospheric pressure towards the capillary located in the anterior section of the mass spectrometer, where they are monitored by its detector (Ifa et al. 2010; Mamun et al. 2020).

DESI-MSI uses two primary ionization mechanisms that are tailored for low- or high-molecular-weight molecules. For low-molecular-weight molecules, ionization is achieved by charge transfer involving electrons or protons. This charge transfer can occur via three pathways: 1) charge transfer between solvent ions and analytes present on the surface; 2) charge transfer between gas-phase ions and the analyte on the surface after the solvent ions have evaporated prior to reaching the sample’s surface; and 3) charge transfer between ions in the gas phase and analyte molecules in the gas phase, which occurs when the sample exhibits high vapor pressure. For high-molecular-weight molecules such as proteins and polypeptides, the presence of multi-charge ions resembling electrospray can be observed. Upon impact with the sample, the charged droplets disperse over a range wider than their own diameter. After dissolving the protein and reflecting, these droplets enter the mass spectrometer and undergo further desolvation.

Similar to MALDI-MSI, DESI-MSI involves three major steps: 1) sample pretreatment, 2) sample testing, and 3) data processing and imaging analysis. During sample pretreatment, sample selection and preparation typically involve slicing of frozen roots, stems, or other tissues. However, for plant rhizome tissues with high water content or delicate surfaces, the purging of spray gas can cause the sample to deform or migrate. In such cases, the preferred treatment method is imprinting on a thin-layer chromatography plate (Conceição et al. 2021). For petals, leaves, and other thin tissues, it is important to ensure a flat surface, which can be achieved by direct blowing or indirect imaging through stamping. If the wax coating on plant leaves affects the ionization efficiency, chloroform immersion can be performed to remove the wax (Li et al. 2011; B. Li et al. 2013). In DESI-MSI analysis, numerous factors influence detection, including solvent type, concentration, and the presence of an additive as well as the application of high voltage to the spray solvent. Additionally, selecting the appropriate purging angle and height is crucial for sample extraction, ensuring optimal reflection of the droplet beam into the mass spectrometer at an ideal angle and improving the response of the compound (Gilmore et al. 2019). Finally, the acquired data are imported into established imaging software (either commercially available or open source), such as MSIReader, Biomap, and HDI, to reveal the target molecule’s distribution within the sample and quantify its abundance (Bennett & Fernandez 2015). Nie et al. obtained the metabolite spectrum of *Isatidis radix* by DESI-Q-TOF/MS and detected many metabolites, including alkaloids, sulfur compounds, phenylpropanoids, nucleosides, amino acids, organic acids, flavonoids, phenols, terpenes, sugars, peptides, and sphingolipids. These compounds were identified with the help of the UNIFI database of *I. radix* and by referring to isotope peaks, reference standards, the literature, and LC–MS/MS data, and the spatial distribution of different metabolites in tissues was visualized. Finally, using multivariate statistical analysis, potential quality markers of *I. radix* were identified, providing a scientific basis for the quality evaluation of Chinese herbal medicine (Nie et al. 2022).

Based on DESI-MSI technology, Zeper Abliz and others developed air flow-assisted desorption electrospray ionization–mass spectrometry imaging (AFADESI-MSI), which enables long-distance ion transport at atmospheric pressure through high-speed air flow in conjunction with solvent removal, thus improving ionization efficiency and analytical sensitivity. This technique expands the space of samples to be detected and enhances the flexibility of operation (X. Meng et al. 2023). DESI technology offers the following advantages: 1) The ionization process is conducted under ambient temperature and pressure conditions. 2) Matrix treatment is unnecessary, thereby circumventing matrix interference in the low-mass range and rendering it suitable for imaging small compounds. 3) This technique can better avoid displacement of the detected molecule caused by the matrix dissolving the sample, thereby ensuring accuracy. 4) Using soft ionization, relatively few fragment ions are produced. Nevertheless, the spatial resolution of DESI is inferior to that of MALDI and SIMS, making it challenging to dissociate protein macromolecules and nonpolar substances, thereby leading to lower sensitivity (Parrot et al. 2018).

### SIMS imaging

SIMS is recognized as the most advanced MSI technique due to its exceptional spatial resolution. This label-free method offers numerous advantages, including high sensitivity, multi-component detection, and imaging at submicron spatial resolution (Strick et al. 2001). SIMS has extensive applications in cell biology, histopathology, biomedicine, and clinical medicine, but its utilization in plant research remains relatively limited. The origins of SIMS can be traced back to the early 20th century when British physicist Thomson first observed the “secondary ray” phenomenon during his collision experiments with metal plates. This phenomenon refers to the neutral particles and positively charged ions produced by ion initiation. In 1936, Aront and Milligan analyzed the energy distribution and ion yield of negatively charged secondary ions resulting from bombardment by positive ion beams using magnetic field deflection. Building upon this work, Herzog and Viehboeck examined the fundamental principles of secondary ions and subsequently developed the initial secondary ion mass spectrometer in 1949. The pioneering efforts of Beninghoven’s research team in the 1970s led to the commercialization of the world’s first SIMS instrument, known as TOF–SIMS (Jabs et al. 1986).

**Fig. 4:**
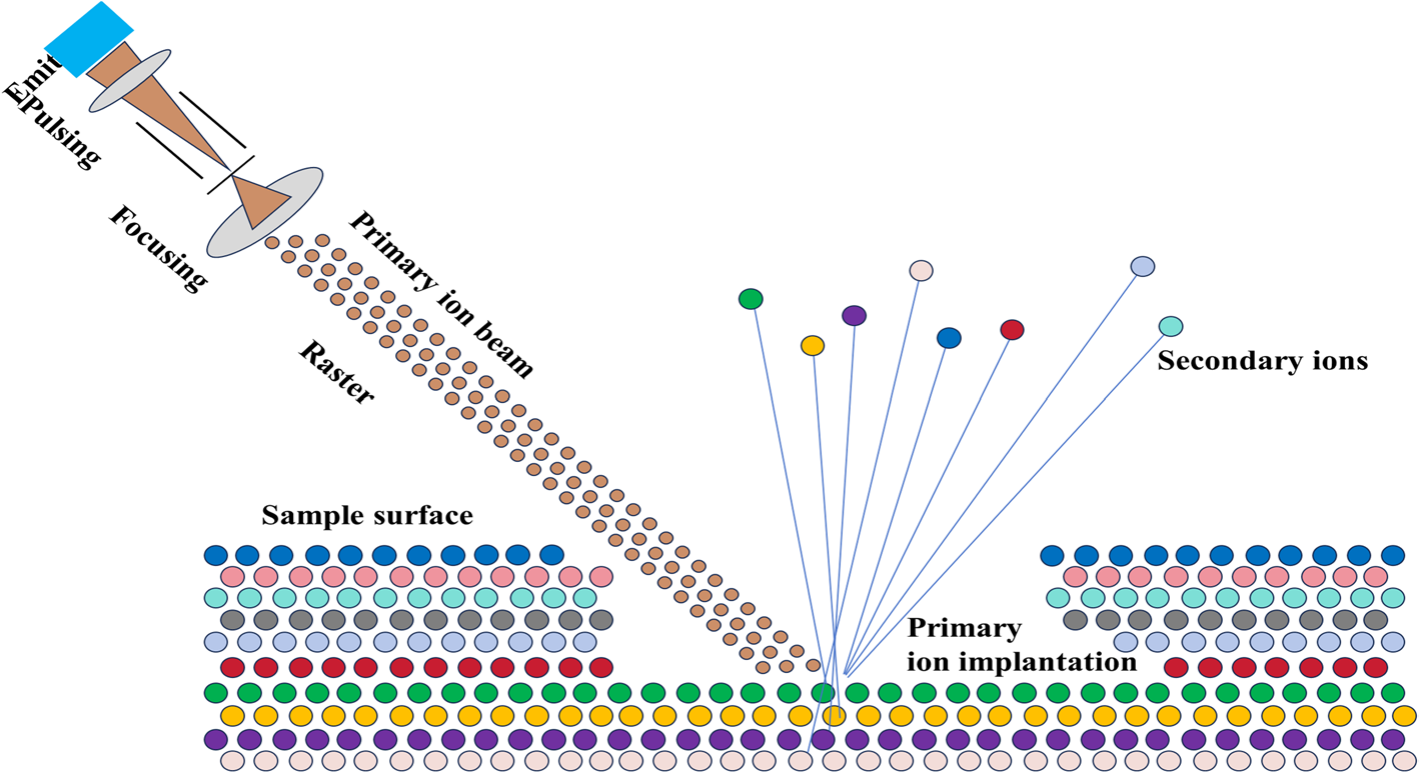
Schematic diagram of secondary ion mass spectrometry (SIMS) imaging

Figure 4 illustrates the fundamental mechanism of SIMS. This technique involves directing a concentrated primary ion beam with high energy toward the sample’s surface. Upon penetration, the primary ions traverse a specific depth and undergo cascading collisions, inducing physical and chemical changes to the sample’s surface atoms. Consequently, secondary ions are sputtered from the surface. These sputtered secondary ions are subsequently separated by a mass analyzer based on their *m*/*z*, ultimately yielding a mass spectrum. A computer captures the ion signal intensity and spatial coordinate data at each pixel and utilizes this information to generate an ion image. SIMS initially employed gallium ion (Ga^+^) and indium ion (In^+^) liquid metal field emission sources as ionization sources. However, due to their elevated excitation energy, these sources caused organic macromolecules to dissociate, thereby restricting the identification of compounds with molecular weights below 200 D. Subsequent advancements in this technology involved using metal cluster ions (such as gold ions (Au_*x*_^+^) and bismuth ions (Bi_*x*_^+^)(*x* ≈ 1–7)) and polyatomic ions (such as sulfur pentafluoride ion (SF_5_^+^) and C_60_^+^) to detect large organic molecules. Metal cluster ions substantially enhance the detection sensitivity of organic macromolecules while preserving submicron image resolution. Polyatomic ions also exhibit sensitivity toward organic macromolecules but display relatively inferior image resolution. The cluster bismuth ion (Bi^3+^) beam is extensively employed in SIMS imaging analysis. Selection of the appropriate ion beam should be based on the specific requirements of the sample under examination (Strick et al. 2001).

The sample preparation and processing methods for SIMS imaging are similar to those for MALDI-MSI and DESI-MSI, and frozen sections are usually used. In general, prepared tissue samples can be analyzed by SIMS imaging without the need for supplemental treatment. Nevertheless, the detection sensitivity of SIMS can be enhanced by applying gold and silver films or chemical matrixes with nanometer thickness to the sample surface. This technique has proven successful for examining biomolecules such as lipids and peptides. The processing of TOF–SIMS data involves data regeneration and information extraction. Data regeneration technology saves all the information detected in each cycle in a computer. As long as the data are read according to the time sequence, the same process as the actual test can be reproduced. Therefore, ions, fragment ions, and molecular ions that were forgotten or ignored in the initial test can be added for analysis during data regeneration. An important benefit of data regeneration technology is that it can obtain data in any selected region of interest and at any selected point in space (surface) and time (depth). Information about the chemical components contained on the surfaces of materials or chemical components of interest is extracted from the test data from TOF–SIMS via fusion into an Orbitrap mass analyzer and multivariate mathematical statistical analysis (such as PCA and deep machine learning) (Frisz et al. 2012; Le Roux & Bengio 2008; Passarelli et al. 2017; Wang et al. 2017).

Building upon the existing SIMS technology, researchers have integrated ultra-high-resolution microscopy with isotope tracing technology to develop nano secondary ion mass spectrometry imaging (Nano-SIMS) technology, which offers superior resolution. The primary high-energy ion beam can be directed toward the sample surface within an ultra-high-vacuum environment, resulting in systematic excitation of the sample material. This enables the generation of quantitative atomic distribution maps for selected elements and their isotopes on the sample surface, allowing simultaneous detection of the distribution of seven elements or isotopes. Based on Nano-SIMS technology, Harmsen et al. employed multiplexed ion beam imaging (MIBI) to attach single-isotope metal labels to antibodies. These labeled antibodies were then used to immunostain formalin-fixed paraffin-embedded tissue sections. The SIMS technique was employed to stabilize the antibody-labeled antigen labeled with metal isotopes, enabling in situ protein imaging. This breakthrough successfully overcame the constraints of detection channels arising from spectral overlap using traditional immunofluorescence technology (Harmsen et al. 2020). Subsequently, Keren et al. substituted the original magnetic field analyzer with a TOF analyzer to establish the MIBI-TOF system. This modification further expanded the capacity to simultaneously analyze multiple channels, enabling imaging of 36 proteins at the same time. Moreover, the imaging resolution was at the subcellular level (Keren et al., 2019). The use of SIMS for analyzing specific biomolecules or marker elements in plant tissue slices is expected to increase due to its exceptional capability for mass spectrometry analysis and its high spatial resolution.

### Summary

The three most common MSI technologies differ in their working principles, resulting in distinct advantages and disadvantages in their practical applications. These differences primarily relate to four components (Table 1): the target substance to be analyzed, sample pretreatment method, spatial resolution, and the mass spectrometry acquisition environment. First, regarding the target substance, SIMS is a high-energy desorption method, which has obvious advantages for element analysis. However, the impact of primary ions can easily damage molecular structures, and the sputtering of secondary particles onto the sample surface is limited by the particle size; therefore, SIMS has a limited detection range and can usually be applied only to small compounds with *m*/*z* below 3000 Da. MALDI involves a milder ionization mode than SIMS and has therefore been widely used to analyze proteins, peptides, phospholipids, and other high-molecular-weight substances. DESI has the characteristics of soft ionization, produces fewer ion fragments, and is suitable for small molecular compounds that are easily ionized, such as organic acids and alkaloids. Second, regarding sample pretreatment, the coating of the surface matrix is indispensable in MALDI-MSI, while DESI-MSI and SIMS imaging do not require a matrix on the sample surface, which reduces the interference from matrix peaks. Third, the spatial resolution of MSI determines the size of the component(s) that can be analyzed and the imaging accuracy. The spatial resolution of SIMS imaging is the highest: The horizontal resolution of Nano-SIMS can reach 50 nm, which is sufficient to support single-cell or even subcellular imaging analysis. The spatial resolution of MALDI-MSI is the next highest, with values generally between 50 and 100 μm. The spatial resolution of MALDI-MSI reported in the literature to date is as low as 1.4 μm, but only when the time for sample scanning was greatly prolonged.

**Table 1:** Comparison of the three most commonly used ionization methods for MSI of plant samples

Ionization type	Ionization source	Applications	Spatial resolution	Conditions
MALDI	IR/UV (laser)	cellulose, hemicellulose, lignin, oligosaccharides, metabolites	~ 1.4 μm	high/medium vacuum or ambient; wide detection range; requires a matrix
DESI	charged corpuscle (solvent)	organic acids, alkaloids, metabolites	~ 20 μm	ambient; no matrix required
SIMS	primary ion beam (ions)	elements, cellulose, lignin, polysaccharides, metabolites	~ 50 nm	high vacuum; high resolution; no matrix required

When used to analyze biological samples, DESI-MSI generally shows a spatial resolution between tens and hundreds of microns, but it can reach 20 μm with an optimized solvent flow rate, capillary diameter, and mass spectrometry scanning speed. The three imaging technologies have different requirements for their mass spectrometry acquisition environment: SIMS requires a high-vacuum environment and is therefore not suitable for analyzing compounds that are unstable in a vacuum. DESI involves open mass spectrometry under atmospheric conditions. MALDI usually requires a vacuum, but the development of AP-MALDI has expanded its application for imaging.

## Applications of MSI technology for botanical research

The biosynthesis and accumulation of secondary metabolites in plants are spatially regulated, and the specific distribution of metabolites in tissues is closely related to their physiological functions. Therefore, it is important to study the tissue distribution characteristics and accumulation of secondary metabolites in plants at different growth stages and in different tissues and organs using MSI technology. These investigations will help us better understand plant secondary metabolite biosynthesis to optimize cultivation and production techniques and to identify and maintain elite germplasm resources. The plants examined, tissue types, MSI methods, and imaged molecules described in this article are summarized in Table 2; the studies are listed in order based on the classification and year of publication. In the following sections, we provide examples of research applications and progress in plant biology research and plant processing using the three MSI technologies.

**Table 2:** Applications of MALDI-MSI, DESI-MSI and TOF–SIMS in plant research

Plant	Tissue type	MSI method	Imaged molecules	Ref.
*Laba garlic*	Whole plants	MALDI-MSI	Sugars, amino acids, organic sulfur compounds, and saponins	(N. Li et al. 2023)
*Taxus chinensis*	Stems	MALDI-MSI	Taxanes	(Yu et al., 2023)
*Peanut*	Seeds	MALDI-MSI	Lipids	(X. Wang et al. 2022)
*Wheat*	Seeds	(AP) SMALDI-MSI	Metabolites	(Righetti et al. 2022)
*Paeonia suffruticosa* and *Paeonia lactiflora*	Roots	MALDI-MSI	Monoterpene and paeonol glycosides, tannins, flavonoids, saccharides, and lipids	(Li et al. 2021)
*Clausena lansium* (*Lour.*)	Fruits, leaves, and stems	MALDI-MSI	Alkaloids, coumarins, sugars, and organic acids	(X. Tang et al., 2021)
*Asclepias curassavica*	Injury site	3D-surface MALDI-MSI	Plant defensive cardiac glycosides	(Dreisbach et al. 2021)
*Wolfberry*	Fruits and seeds	MALDI-MSI	Metabolites such as choline, betaine, hexose, and sucrose	(Zhao et al. 2021)
*Maize*	Roots	AP-SMALDI-MSI	Metabolism	(Righetti et al. 2021)
*Panax notoginseng*	Roots	MALDI-MSI	Metabolites	(Sun et al. 2021)
*Lepidium meyenii*	Roots	MALDI-MSI	Secondary metabolites (alkaloids)	(Mi et al. 2020)
*Grain sorghum*	Seeds	MALDI-MSI	Dhurrin	(Montini et al. 2020)
*Barley*	Seeds\roots	MALDI-MSI	Lipids\salt stress	(Gupta et al. 2019); (Sarabia et al. 2018)
*Aquilaria sinensis*	Wood slices	MALDI-MSI	Natural products	(Kuo et al. 2019)
*Ginkgo biloba*	Leaves	MALDI-MSI	Lipids	(B. Li et al. 2018)
*Hypericum perforatum L*	Roots	MALDI-MSI	Xanthone, plant defense	(Tocci et al. 2018)
*Panax ginseng*	Roots	MALDI-MSI	Ginsenosides	(Dueñas et al. 2017; S. J. Wang et al. 2016)
*Panax ginseng*, *Panax quinquefolius* and *Panax notoginseng*	Roots	MALDI-MSI	Saponins	(S. J. Wang et al. 2016)
*Wheat*	Oilseed rape seeds, wheat rachis, wheat seeds, wheat stem bases, and rice roots	MALDI-MSI	Lysophosphatidylcholine, polysaccharides, plant defense	(Bhandari et al. 2015)
*Rice*	Roots	Nano-PALDI-MS	Plant hormones/crop development and stress response	(Shiono & Taira 2020)
*Gelsemium elegans*	Leaves, stems, and roots	DESI-MSI	Multiple alkaloids	(Wu et al. 2022b)
*Almonds, hazelnuts, cashews, walnuts, peanuts, peach seeds, bitter almonds, and Chinese dwarf cherry*	Seeds	DESI-MSI	Lipids, glyceride, glycerophosphate, and fatty acids	(Hou et al. 2022)
*Tea*	Leaves	DESI/PI) MSI	Metabolites such as caffeine and theanine	(L. Wu et al. 2022)
*Ocotea spixiana*	Twigs	DESI-MSI	Benzylisoquinoline and aporphine alkaloids	(Conceicao et al. 2020)
*Camellia sinensis*	Leaves and stems	DESI-MSI		(Liao et al. 2019)
*Mentha* × *piperita L*	Leaves	DESI-MSI	Flavonoids	(Freitas J, Vendramini P, Melo J, et al. 2019)
*Rauvolfia tetraphylla L*	Stems, leaves, roots, and fruits	DESI-MSI	Alkaloids	(Mohana Kumara et al. 2019)
*Psychotria prunifolia (Kunth) Steyerm and Palicourea coriacea (Cham.) K. Schum*	Leaves	DESI-MSI	Alkaloids	(Kato et al., 2018)
*Pyrenacantha volubilis Hook*	Seeds and fruits	DESI-MSI	Alkaloids (Camptothecin)	(Kumarappa et al., 2017)
*Coffee bean*	Endosperm	DESI-MSI	Chlorogenic acids and sucrose	(Garrett et al. 2016)
*Pyrenacantha volubilis*	Leaves and stem	DESI-MSI	Pesticides	(Gerbig et al. 2015)
*Fern*	Leaves	DESI-MSI	Arsenic compounds	(de Abreu et al. 2013)
*Seaweed*	Whole plants	DESI-MSI	Chemical defense	(Lane et al. 2009)
*Seaweed*	Whole plants	DESI-MSI	Chemical defense	(Esquenazi et al. 2009)
*Cordyceps sinensis*	Whole plants	TOF–SIMS	Secondary metabolites	(Q. B. Liu et al. 2022)

### Applications of MSI in plant breeding

MSI can provide visual information about the spatial distribution and transport of metabolites in plant tissues, thereby providing guidance for breeding plants with improved economic or ecological properties. Righetti et al. used MSI to analyze the grains of common wheat (*Triticum aestivum*) and durum wheat (*Triticum durum*) contaminated by the mycotoxin deoxynivalenol (DON) and visualized the tissue-specific distribution of metabolites and lipid components related to *Fusarium* invasion and DON accumulation. The authors detected diacylglycerol DG (33:4) and DG (33:3) in the contaminated wheat seeds, with the DGs concentrated in the outer layers of the seed. Moreover, DGs were found only in contaminated wheat samples but not in healthy samples, suggesting that DG accumulation may be caused by membrane degeneration by the pathogenic fungus (Righetti et al. 2022). Zhao et al. used MSI to visualize the spatial distribution of endogenous molecules in wolfberry (*Lycium barbarum* L.) fruit tissue. Choline, betaine, and citric acid were evenly distributed throughout the fruit at all stages of growth, hexose was mainly distributed in endocarp and pulp tissue, and sucrose was mainly concentrated in seeds. Furthermore, over the course of fruit development, the signal intensity of citric acid gradually weakened, while the signal intensities of choline, betaine, hexose, and sucrose gradually increased (Zhao et al. 2021). Bhandari et al. used high-spatial-resolution MSI technology to compare and analyze the contents of oilseed rape (*Brassica napus*) seeds during germination and maturity. The concentration of spermidine conjugate was related to the development of the hypocotyl/radicle: During seed germination, the cyclic spermidine conjugate was transferred from the hypocotyl/radicle to the budding young root. Trichlorocaffeoyl spermidine showed the same trend (Bhandari et al. 2015). These studies demonstrated that MSI is an effective research tool for analyzing the defense mechanisms of plants against fungal invasion, revealing the tissue-specific distribution of important secondary metabolites during different stages of plant growth. Analyzing the roles of these metabolites in plant growth and development would help optimize plant cultivation and production and enhance germplasm improvement.

### Applications of MSI technology for studying plant growth and development and for quality identification

The locations of secondary metabolites in plant tissues are closely related to their physiological functions. MSI can reveal the biosynthesis and metabolic pathways as well as the spatial distribution of components in plants in situ, providing a powerful tool to study the distribution and accumulation of active components in plants. DESI-MSI is frequently used in plant research. Hou et al. used desorption electrospray ionization combined with ion mobility-quadrupole-time-of-flight (DESI-IM-QTOF) MSI technology to study the spatial distribution of lipids in seeds from three plant species used in Chinese medicine (peach [*Prunus persica*], bitter almond [*Prunus amygdalus* var. *amara*], and plum [*Prunus mume*]) and five types of edible nuts (almond [*Prunus dulcis*], hazelnut [*Corylus avellana*], cashew [*Anacardium occidentale*], walnut [*Juglans regia*], and peanut [*Arachis hypogaea*]) in positive and negative ion modes. The authors also analyzed the accumulation and distribution of glyceride, glycerophosphate, fatty acids, and other lipid components (Hou et al. 2022).

Wu et al. designed a desorption electrospray ionization/post-photoionization (DESI/PI) MSI platform combined with porous polytetrafluoroethylene (PTFE) imprinting technology to analyze tea (*Camellia sinensis*) leaves. Caffeine was mainly distributed in the midveins of leaves, and theanine was enriched in the petiole and extended into the midveins and leaf tail. They also analyzed the spatial distribution of catechin, an important flavonoid metabolite (L. Wu et al. 2022). Liao et al. studied the spatial distribution of polyphenols in tea tissues by DESI-MSI. Epicatechin, gallic acid, catechin gallic acid, epigallocatechin, gallate ester, and gallocatechin were evenly distributed on both sides of leaves, while epicatechin, catechin, epigallocatechin, and gallocatechin were also distributed near the vein. However, L-theanine was mainly detected in roots, and L-theanine and valine were mainly distributed in cross sections of outer roots (Liao et al. 2019).

Wu et al. used DESI-MSI to analyze the distribution of bioactive alkaloids in different tissues of *Gelsemium elegans* at different stages of growth. The authors isolated 23 alkaloids from root, stem, and leaf tissues at the seedling stage and 19 alkaloids at the mature stage. Among these alkaloids, 16 were found in the vascular bundles of roots in mature *G. elegans* plants, 15 were detected in the piths of mature stems, and 2 were found in the stem epidermis. The authors also investigated the diffusion and transfer paths of other alkaloids in *G. elegans* throughout its life cycle (Z. H. Wu et al. 2022). Freitas et al. used DESI-MSI to study the spatial distribution of the major flavonoids in the leaves of *Mentha* × *Piperita* L. and revealed three major metabolic pathways of these flavonoids: the naringin, luteolin, and apigenin pathways (Freitas et al., 2019). Garrett et al. used DESI-MSI to analyze and quantify the distribution of various compounds in different tissues of coffee plant (*Coffea* spp.). Chlorogenic acid, an active substance related to flavor and ultraviolet light protection in coffee beans, was unevenly distributed in the endosperm. Quinic acid and feruloyl quinic acid were mainly distributed in the hard endosperm, and caffeine quinine had high signal intensity mainly in the soft endosperm (Garrett et al. 2016). In addition, using alkaloids that are easy to ionize, DESI-MSI was also used to analyze the spatial distribution of different alkaloids in medicinal plant samples rich in alkaloids, such as *Ocotea spixiana* (Conceicao et al. 2020), *Rauvolfia tetraphylla* L. (Mohana Kumara et al., 2019), *Pyrenacantha volubilis* (Kumarappa et al., 2017), and *Psychotria rubra* (Lour.) Poir (Kato et al., 2018). These studies on the metabolism, transport, and accumulation of these alkaloids provide guidance for improving the medicinal value of these plants.

MALDI-MSI has higher spatial resolution than DESI-MSI and SIMS imaging and is also widely used in research on plant growth and development, allowing investigations at the single-cell level. Yu et al. used MALDI-MSI to create a distribution map of taxanes in young stems of *Taxus chinensis*, which revealed that these compounds accumulate in a tissue-specific manner. The authors determined that 10-deacetyl paclitaxel is evenly distributed throughout the stem, whereas 10-deacetylbaccatin and baccatin are mainly concentrated in the endodermis and phloem, and taxol specifically accumulates in the outer cortex. Gene expression maps of the major cell types in *T. chinensis* showed that *ctg8816_gene.2*, *ctg7747_gene.2*, and the taxadiene synthase gene are mainly expressed in inner cortex cells, *ctg2120_gene.10* and *ctg887_gene.19* are mainly expressed in xylem parenchyma cells, and *ctg5026_gene.32* is expressed in outer cortex cells (Yu et al., 2023).

Wang et al. studied the contents and spatial distribution of lipid substances and other key metabolites in the seeds of three peanut varieties by MALDI-MSI and identified 103 metabolites, including 34 lipid components. Among these, lysophosphatidylcholine, phosphatidylethanolamine, and phosphatidylcholine are mainly distributed in seeds. However, the metabolic characteristics of phosphatidic acid and triacylglycerols differ significantly between different tissues of peanut seeds, suggesting that the uneven spatial distribution of metabolites is closely related to the functional differences of specific tissues (X. Wang et al. 2022). X. Tang et al. studied the tissue-specific distribution characteristics of metabolites in *Clausena lansium* (Lour.) by MALDI-MSI and found that 3- methylcarbazole and murrastinine are mainly distributed in fruits, murrayanine and heptaphylline are mainly distributed in pulp tissue, and girinimbine only exists in the outer skins of fruit stones and the stem epidermis. The tissue-specific distribution and accumulation of coumarins and hexoses in different tissues of this plant have also been analyzed (X. Tang et al., 2021).

Sun et al. used MALDI-MSI to analyze the metabolites in different organs (rhizomes, taproots, branched roots, and fibrous roots) and different tissues (phloem, xylem, medulla, and cork tissue) of *Panax notoginseng*. Different saponins and amino acids showed different distribution patterns. For example, the highest levels of notoginsenoside R_1_ were present in phloem and xylem tissue, amino acids were mainly distributed in medulla and phloem tissue, and notoginsenoside and citric acid were mainly distributed in taproots (Sun et al. 2021). Zhao et al. used MALDI-MSI to analyze the spatial distribution of endogenous molecules in *Lycium barbarum* fruit tissues and analyzed the correlation between the in situ distribution of components and their physiological functions (Zhao et al. 2021). Mi et al. studied the distribution of imidazole alkaloids and benzyl glucosinolates in cross sections of maca (*Lepidium meyenii* Walp) roots by MALDI-MSI. Two different types of imidazole alkaloids had similar distribution patterns in the root cortex and outer cortex, while benzyl glucosinolates were mainly concentrated in the medulla of the root (Mi et al. 2020). Kuo et al. used MALDI-MSI and mass spectrometry–based molecular network fusion technology to explore the spatial distribution characteristics of biomolecules in wood slices of *Aquilaria sinensis* and established a method for studying natural products in this plant. The authors analyzed the spatial distribution patterns of more than 30 *A. sinensis* natural products in a single sample (Kuo et al. 2019). Wang et al. used MALDI-MSI to detect the tissue-specific distribution of saponins in *Ginseng Radix et Rhizoma*, *Notoginseng radix*, and *Panax quinquefolius* and constructed a metabolite distribution map to distinguish the above three metabolites (S. J. Wang et al. 2016). Thus, MALDI-MSI has been successfully used to study metabolite distribution in a wide variety of plants.

Although SIMS has not yet been employed in plants, an elegant example of its application in biological systems comes from Liu et al. (2022), who used SIMS imaging for in situ chemical analysis and imaging of bioactive compounds in natural and cultured *Cordyceps sinensis*–infected caterpillars. Most components were distributed uniformly in the infected caterpillars, but triacylglycerol, diacylglycerol, monoacylglycerol, and fatty acids were only found outside the digestive tract (Q. B. Liu et al. 2022). Quantitative analysis of the detected components showed that unlike fatty acids, glycerides, and glycerophosphates, the proportions of amino acids, nucleosides, monosaccharides, sphingolipids, sterols, and other components were similar between natural and cultured *C. sinensis–*infected caterpillars. These findings provide visual data support for research on the quality of cultivated medicinal materials and the substitution of natural precious medicinal materials with these materials. The above example illustrates how SIMS could be used to study the spatial distribution of secondary metabolites in plants, analyze plant metabolic pathways, and investigate the accumulation of bioactive compounds in the tissues of medicinal plants to identify promising medicinal materials and improve the economic value of medicinal plants.

### Applications of MSI technology to plant stress tolerance research

The biosynthesis of secondary metabolites in plants generally increases when plants are exposed to adverse environmental conditions. Several studies have examined the responses of various plants to stress using mass spectrometry to elucidate the defense mechanisms they employ. Righetti et al. used MSI to visualize the grains of common wheat and durum wheat contaminated with DON. Hydroxycinnamic acid amides with antifungal activity were detected in the cuticles of infected wheat seed coats, which passively defended the plant against pathogen attack on the seed surface. Additionally, certain components accumulated rapidly upon fungal infection, thereby enhancing plant resistance to pathogenic fungi (Righetti et al. 2022).

Righetti et al. employed atmospheric pressure scanning microprobe matrix-assisted laser desorption/ionization–mass spectrometry imaging (AP-SMALDI MSI) to investigate the spatial distribution and metabolic destiny of aflatoxin B1 (AFB1) and its potential modified form (occult mycotoxin) in maize (*Zea mays*) plants that were infected with fungi such as Aspergillus, as well as the distribution of plant stress metabolites in various tissues, organs, and cells. The authors determined that AFB1 accumulation hindered chlorophyll and anthocyanin biosynthesis in maize roots, potentially influencing the phytohormone response, altering chlorophyll metabolism, and suppressing anthocyanin biosynthesis by inhibiting cytokinin signaling. Additionally, chlorophyll biosynthesis decreased in the roots of plants treated with AFB1, and anthocyanins involved in defense were detected only in healthy maize plants, suggesting that AFB1 weakens the maize defense system (Righetti et al. 2021).

Dreisbach used auto-focusing three-dimensional AP-SMALDI MSI technology to examine the defense metabolites present in the leaves of *Asclepias curassavica* L*.* The authors determined that latex serves as an integral component of the plant’s chemical defense against tissue damage caused by phytophagous insects. Following leaf damage, the rate of milk flow to the wound intensified, leading to the accumulation of defensive substances in the affected areas. Additionally, simulation of the insect predation strategy (pulse cutting) showed that fewer toxic metabolites accumulated at the distal end of the cut wound. Numerous unidentified ion signals similar to cardiac glycosides showed the same spatial distribution pattern, revealing the diversity of defensive substances in *A. curassavica* (Dreisbach et al. 2021).

Montini employed targeted metabolite analysis in conjunction with MALDI-MSI to investigate the accumulation and distribution of dhurrin, a key chemical defense compound that releases toxic hydrogen cyanide upon tissue disruption in sorghum (*Sorghum bicolor*). The authors measured the levels of dhurrin, its recycling products, and key general metabolites across four distinct sorghum varieties during grain imbibition, germination, and early seedling development. Little or no dhurrin or recycling products were present in dry grains, but their de novo biosynthesis started immediately after water uptake. Within a 24-h period, this led to the rapid accumulation of dhurrin, accompanied by an increase in the concentration of free amino acids, indicating the initiation of germination. Additionally, the authors examined the cumulative trajectory and final concentrations of dhurrin in the sorghum varieties, which also influenced the composition and quantity of recycling products and free amino acids. Notably, dhurrin was predominantly enriched in germinated embryos, pointing to its role in safeguarding nascent tissues against herbivory (Montini et al. 2020).

Shiono et al. used nanoparticle-assisted laser desorption/ionization–mass spectrometry to image and analyze various plant hormones in rice (*Oryza sativa*) roots. High levels of abscisic acid and cytokinins were detected in the outer parts of the root, whereas lower levels were present in the stele. Brassinosteroid, salicylic acid, and 1-aminocylopropane-1-carboxylic acid were widely distributed throughout the root, while indole acetic acid was distributed in the epidermis, cortex, and stele (Shiono & Taira 2020). In a separate study, Gupta et al. employed MALDI-MSI and μ-X-ray fluorescence (μ-XRF) spectroscopy to investigate the temporal and spatial distribution of metabolites and elements during early germination of barley (*Hordeum vulgare*) seeds under salt stress. Additionally, the authors analyzed over 200 lipid compounds across seven categories (fatty acyls, glycerolipids, glycerophospholipids, sphingolipids, prenol lipids, sterol lipids, and polyketides). The spatial distribution of these compounds was influenced by genotype (Mundah or keel), imbibition time (0–72 h), and treatment (control or salt stress) (Gupta et al. 2019; Sarabia et al. 2018). Tocci et al. employed MSI to investigate the distribution characteristics of xanthone in the roots of *Hypericum perforatum*. Xanthone biosynthesis and accumulation occurred in the outer cortex and inner cortex of the root, and it was not translocated to other regions. These results indicate that the outermost and innermost layers of the root cortex not only control the storage and directional transport of water and nutrients, but also serve as the sites of biosynthesis and accumulation of the secondary metabolite xanthone, which functions as a chemical barrier against soil-borne pathogens and nematodes (Tocci et al. 2018).

Li et al. used MALDI-MSI to analyze the spatial distribution of carbohydrates, phospholipids, and chlorophyll a in *Ginkgo biloba* leaves. The authors detected two different lipid distribution patterns: Most lipid components were distributed in the mesophyll, whereas some toxic phenolic lipid components were found only in the secretory cavities, allowing them to be released when the leaves are crushed to protect the plant from herbivory (B. Li et al. 2018). Bhandari et al. used high-resolution MSI to study metabolites in the spike rachis, stem base, and seeds of wheat, finding that lysophosphatidylcholine in the spike rachis and stem base help prevent pathogen infection by participating in the biosynthesis of antifungal peptides, defensive signaling molecules, disease resistance signaling molecules, and other substances. However, polyhexoses were distributed uniformly in the endosperm of healthy and *F. graminearum*–infected wheat seeds, and metabolites related to pathogens were distributed in the bran of diseased seeds (Bhandari et al. 2015). Finally, Esquenazi and others used DESI-MSI to combine natural medicinal chemistry with ecological methods, revealing the surface-mediated antifungal chemical defense mechanism of tropical seaweed (Esquenazi et al. 2009; Lane et al. 2009). These studies using in situ MSI to analyze the production and accumulation of defensive secondary metabolites in plants faced with biotic and abiotic stress provided experimental evidence for the mechanisms underlying stress resilience in plants.

### Applications of MSI technology in plant processing research

Li et al. combined MSI with mass spectrometry to analyze the in situ changes in the levels of major low-molecular-weight compounds (e.g., sugars, amino acids, organic sulfur compounds, and saponins) in tissues during the greening of Laba garlic. The levels of organic sulfur compounds gradually decreased, and these compounds were converted into pigment components during processing. Arginine, the main amino acid in Laba garlic, was also involved in this color transformation process (N. Li et al. 2023). X. Wang et al. studied the spatial distribution of lipid substances and key metabolites in different peanut varieties using MALDI-MSI technology. Phosphatidylcholine (PC), lysophosphatidylcholine (LPC), and phosphatidylethanolamine (PE) were mainly detected in seeds, whereas TG (triglyceride) and PA (phosphatidic acid) showed highly heterogeneous distribution patterns that appeared to be related to the tissue-specific metabolic pathways of lipid biosynthesis (X. Wang et al. 2022).

Bin Li et al. analyzed roots of the medicinal plants *Paeonia suffruticosa* and *Paeonia lactiflora* by MALDI-MSI. The cork layers of the roots of both species were rich in various bioactive components, suggesting that it is necessary to preserve the cork layer as much as possible during production and processing to avoid the outflow of their active components, such as paeoniflorin and paeoniflorin (B. Li et al. 2021). Sun et al. used MALDI-MSI to analyze the temporal and spatial changes in metabolite levels in *Panax notoginseng* roots during the cooking process. Cooking led to a gradual decrease in the content of notoginsenoside, which would reduce the hemostatic effect of notoginseng. However, cooking also increased the contents of arginine, glutamic acid, and some low-molecular-weight organic compounds. This information is useful for evaluating the theoretical considerations associated with cooking *P. notoginseng* in traditional Chinese medicine practices (Sun et al. 2021). Overall, the above work demonstrates the successful application of MSI to provide effective guidance for quality control and method selection in plant processing by analyzing the temporal and spatial variation in components before and after processing.

### Applications of MSI technology to other types of botanical research

MSI technology is increasingly being used to detect pesticide and heavy metal residues in plants as a means of comprehensive plant quality control. Gerbig et al. used mass spectrometry to detect the distribution of two pesticides in cotyledons of *Castanopsis fargesii* following their application to the soil*.* The two pesticides, which had different polarity and solubility levels, were unevenly distributed in leaves, demonstrating that pesticides from soil can be effectively detected in leaves several weeks after pesticide application (Gerbig et al. 2015). De Abreu et al. used DESI-MSI and LC-ICP-MS to analyze the temporal and spatial variation in arsenic levels in different chemical forms in fern fronds and developed specific methods to avoid reciprocal transformation of different chemical forms of arsenic during frond preparation (de Abreu et al. 2013). Finally, Wu et al. used the “gold nano-paper” research strategy combined with MSI technology to analyze the transport mechanisms of targeted pesticides in plants. The authors visually demonstrated the dynamic transport processes of pesticides, from their crossing of the plasma membrane with the aid of transports to their translocation from the top to bottom of the stem through the phloem, diffusion to the xylem, and enrichment at the edges of leaves (X. Wu et al. 2020).

## Summary and future prospects

Over the past two decades, the progressive development and refinement of MSI technology have led to its increasingly widespread use in the field of botany. To date, this technology has been successfully used to elucidate dynamic patterns of metabolite accumulation, biosynthesis pathways, and correlations between the positions and functions of metabolites in numerous plant species, thereby playing an increasingly important role in elucidating the physiological and ecological transformations of plants. Notably, however, low spatial resolution remains a significant constraint that impedes the further utilization of MSI. To address the issue of spatial resolution, researchers have made significant advances in instrumentation, including the development of the needle-tip enhanced desorption mass spectrometer (Liang et al., 2017), near-field desorption imaging mass spectrometer (Yin et al. 2019), and microlens fiber laser desorption ionization mass spectrometer (Y. Meng et al. 2020). These instruments have enabled the acquisition of high-resolution images to detect potassium salt residues with a lateral resolution of 50 nm (Liang et al., 2017). Additionally, selecting the appropriate type of mass spectrometry based on specific research objectives is crucial for ensuring the optimal imaging quality and accuracy of mass spectrometry results. For instance, differentiation between glycyrrhizic acid (*m*/*z* 861.36676) and glycyrrhizin G2 (*m*/*z* 861.38721) can be achieved through high-resolution MSI, but not low-mass-resolution MSI (Li et al. 2014). Consequently, enhancing the spatial resolution and quality of MSI data is of paramount importance for its broader use in botanical studies, which could position this method as the leading tool for spatiotemporal analysis of plant components, including pesticides, in the future.

Currently, the application of MSI in botany faces several practical challenges. One such challenge is the limited accuracy of identifying metabolites solely based on *m*/*z* values and peak intensity. Consequently, auxiliary identification methods, such as MS/MS or LC–MS/MS detection, are often required, increasing the operational complexity and time needed for analysis (Ellis et al. 2018; Feldberg et al. 2018). Additionally, MSI typically exhibits lower molecular coverage than LC–MS or GC–MS due to the absence of chromatographic separation, which restricts the number of ions available for compound resolution within a given space and time, making it impossible to distinguish isomers (Dueñas et al. 2019; O'Neill et al. 2022). Furthermore, the utilization of the absolute pixel ratio for quantitative analysis in MSI poses a significant obstacle due to the occurrence of localized biochemical and anatomical variations, which can result in disparate desorption and ionization efficiencies of specific ions from the sample surface (Rzagalinski & Volmer 2017; Unsihuay et al. 2021). Moreover, conventional high-performance MSI platforms yield extensive, intricate high-dimensional data, amounting to several gigabytes per experiment, which presents challenges in the analysis and dissemination of MSI data.

These challenges have inspired the continuous improvement of MSI techniques. Integrating MSI, MS/MS with stable isotope labeling, and MALDI-2 significantly enhanced the efficacy and dependability of metabolite identification. Moreover, the amalgamation of diverse chemical derivatives enhanced the ionization efficiency and molecular coverage of MSI (Dueñas et al. 2019). MSI’s inability to accurately differentiate isomers can be resolved by integrating MSI with ion mobility spectrometry (IMS), the Paternò-Büchi reaction, epoxy-oxidation, ultraviolet photolysis, ozone-induced dissociation, ion–ion reaction, or electron induction techniques (Dong & Aharoni 2022). Potential ways to improve the reproducibility of the results of MSI include enhancing matrix deposition technology and standardizing MSI data across multiple research centers for collaborative research (Boskamp et al. 2021; Ly et al. 2019; Tressler et al. 2021). In terms of data sharing, the emergence of open-source MSI and the associated BASTet (Berkeley Analysis and Storage Toolkit) code library offer valuable avenues for sharing and replicating the results of MSI analysis. The establishment and improvement of the METASPACE, a community-populated knowledge base of spatial metabolomes from health and disease annotation platforms, have notably accelerated data sharing while enhancing operational efficiency and the verification of results (Dong & Aharoni 2022).

In the future, MSI technology could be integrated with other research methodologies to address a broader range of fundamental issues in botanical research. For instance, combining MSI technology and genomics could facilitate the spatial localization and identification of functional genes. Additionally, the fusion of MSI technology with transcriptomics or protein genomics analysis could help elucidate and visualize the metabolic pathways of enzymes, enabling the precise co-localization of metabolites, enzymes, and expressed genes, thereby enhancing plant breeding through genetic engineering (Cabral & Ifa 2015).

Furthermore, the integration of MSI technology with isotope mass spectrometry and plant tissue culture techniques enables the dynamic analysis of pathways for the biosynthesis and accumulation of active constituents in medicinal plants. Building upon this foundation, the identification of key biological mechanisms governing the enrichment of active constituents in medicinal plants could be facilitated by examining the accumulation patterns of in situ components and leveraging insights provided by spatial transcriptomics technology. This approach could enhance the quality and economic value of medicinal plants.

Consequently, the utilization of MSI technology holds immense potential for various aspects of medicinal plant breeding and analysis of the distribution and biosynthetic pathways of metabolites, plant defense compounds, pesticide residues, heavy metal transport pathways, and the physiological and ecological responses of medicinal plants. This technology has already contributed to the advancement, utilization, and preservation of plants and holds great potential for further development and application.

## Data Availability

No datasets were generated or analyzed during the current study.
